# Evaluation of prognostic factors effect on survival time in patients with colorectal cancer, based on Weibull Competing-Risks Model

**Published:** 2017

**Authors:** Soraya Moamer, Ahmadreza Baghestani, Mohamad Amin Pourhoseingholi, Nastaran Hajizadeh, Farzaneh Ahmadi, Mohsen Norouzinia

**Affiliations:** 1 *Department of Biostatistics,Faculty of Paramedical Sciences, Shahid Beheshti University of Medical Sciences, Tehran, Iran*; 2 *Physiotherapy Research Centre, Department of Biostatistics, Faculty of Paramedical Sciences, Shahid Beheshti University of Medical Sciences, Tehran, Iran*; 3 *Gastroenterology and Liver Diseases Research Center, Research Institute for Gastroenterology and Liver Diseases, Shahid Beheshti University of Medical Sciences, Tehran, Iran*; 4 *Basic and Molecular Epidemiology of Gastrointestinal Disorders Research Center, Research Institute for Gastroenterology and Liver Diseases, Tehran, Iran*

**Keywords:** Survival analysis, Competing risks, Colorectal cancer, Weibull model

## Abstract

**Aim:**

The aim of this study was to assess the association between survival of patients with colorectal cancer and prognostic factors in a competing risk parametric model using Weibull distribution.

**Background:**

The prognosis of colorectal cancer is relatively good in terms of survival time. In many prognostic studies, patients may be exposed to several types of competing events. These different causes of death are called competing risks.

**Methods:**

Data was recorded from 372 patients with colorectal cancer who registered in the Institute for Gastroenterology and Liver Diseases, Shahid Beheshti University of Medical Sciences (Tehran, Iran) from 2004 to 2015 in a retrospective study. Analysis was performed using competing risks model and Weibull distribution. Software used for data analysis was R, and significance level was regarded as 0.05.

**Results:**

The result indicated that, at the end of follow-up, 111 (29.8%) deaths were from colorectal cancer and 14 (3.8%) deaths were due to other diseases. The average body mass index (BMI) was 24.61(SD 3.98). The mean survival time for a patient in 372 was 62.05(SD 48.78) month with median equals to 48 months. According to competing-risks method, only stageIII (HR, 1.69; 95% CI, 1.246-2.315 ), stageIV( HR, 4.51; 95% CI,2.91-6.99 ) and BMI( HR, 0.96; 95% CI, 0.96-0.975) have a significant effect on patient’s survival time.

**Conclusion:**

This study indicated pathologic stage (III,IV) and BMI as the prognosis, using a Weibull model with competing risks analysis, while other models without the competing events lead to significant predictors which may be due to over-estimation.

## Introduction

Cancer is a major health problem worldwide (1). Colorectal cancer (CRC) includes large bowel cancer (colon cancer) and cancer of the back passage (rectal cancer or cancer of the rectum) (2). It was the second leading cause of cancer mortality among people of the United States in 2015, with 132 700 new cases and 49 700 deaths (3). The rate of CRC incidence is higher in economically developing countries compared to economically developed countries, also its incidence is higher in men than women (4). Based on the reports of the World Health Organization(WHO), CRC incidence rates have rapidly increased in several areas with low risk, including Asian countries such as China, Japan, South Korea and Singapore that have experienced a 2-4-fold increase in the incidence of CRC during the recent decades (5). The survival rate of CRC is also lower in developing countries compared to developed countries (6).

In Iran, Colorectal cancer is the third most common cancer. The increasing incidence of colorectal cancer (CRC) in the past decades in Iran has made it a major public health problem (7). According to the Iranian Annual National Cancer Registration Report, CRC is the third most common cancer in Iranian women and fifth common cancer in men (8). So Assessment of factors which affect this cancer is important for prolonging the patient’s survival time.

The prognosis of CRC is relatively good in terms of survival time (9) . In survival analysis, competing risks are events that their occurrence precludes the outcome of interest (10). Consider a study with oncological mortality as the outcome; a patient dying of coronary disease (the competing event) cannot also die of cancer (the outcome of interest). These different causes of death are called competing risks (11). Different non-parametric, semi-parametric and parametric models can be used for survival estimation in the presence of competing risks. The parametric model is studied assuming that the competing risks follow different lifetime distributions such as exponential, gamma, and Weibull. The exponential distribution can have only a constant hazard, so it has a limitation to model real data.The Weibull distribution is commonly used for survival analysis with monotone hazard(13).

Therefore, in this study, because subjects’ death may be due to colorectal cancer or other causes, so the purpose of this study was to evaluate the association between survival of patients with colorectal cancer and prognostic factors in a competing risk parametric model using Weibull distribution. 

## Materials and Methods

 Data for 372 patients with colorectal cancer were collected from patients who have registered in the Cancer Registry Center of the Research Center of Gastroenterology and Liver Disease, Shahid Beheshti University of Medical Sciences from 2004 to 2015 were used in this study and their survival situation was identified. 

 Deaths of subjects were confirmed via contact with their families and relatives, which is an official plan of the cancer department in Research Institute of Gastroenterology and Liver Disease and each year one or two times the last situation of all registered patients is followed by telephone contact. In some cases with skeptical information, the telephone contact is repeated to assure the accuracy of information regarding new data. Causes of death were grouped into two competing events: death from colorectal cancer and death from all other causes. In this study, the factors that were examined in patients with colorectal cancer included sex, age at diagnosis (in years), body mass index (BMI), and cancer stage at diagnosis that was categorized into four stages (I–IV), according to the American Joint Committee on Cancer (AJCC) stage classification (12). In this study, since death of subjects may be due to colorectal cancer or other causes, the competing risks model was used for analyzing data. Data analysis was performed using the Weibull competing risks model.

 In analyzing the competing risks data for each person there existed one type of failure (type of event:) in addition to the failure time (survival time). The failure time (T) was assumed to be a continuous and positive random variable, while the failure cause (k) took values in the finite set (k ≥2) j=1,…,k. For colorectal cancer data, the first cause of failure (death due to colorectal cancer, j=1) was considered as the main event in survival model, and the second cause (death due to other events, j=2) was considered as the competing risks. We assumed that survival time of each competing risks has four parameter log-logistic distributions. The survival function for each of the competing risks (the cause of failure of j) is defined as follows:

*S*_j_ *( t; *𝜆_j _*,*$p_{j}$*) = exp*$\left(-\lambda_{j}t^{p_{j}}\right)$*            j=1,2*

In order to assess the effects of sex, age at diagnosis, BMI and tumor stage on the survival time, we defined the scale parameter 𝜆j in the form of a linear combination of covariate.

 Also Weibull model analysis was done without considering the competing risks. Acceptance of Weibull model can be checked via graphical assessments (13).

 Data for continuous variables were reported as the mean ± standard deviation. Discrete values were reported as the number with the corresponding percentage. Hazard ratios (HRs) were reported as point estimates with 95% confidence intervals (95% CIs). All statistical analyses were performed using R statistical software (version 3.0.3) and statistical significance was assumed for P-values<0.05.

## Results

**Figure 1:**
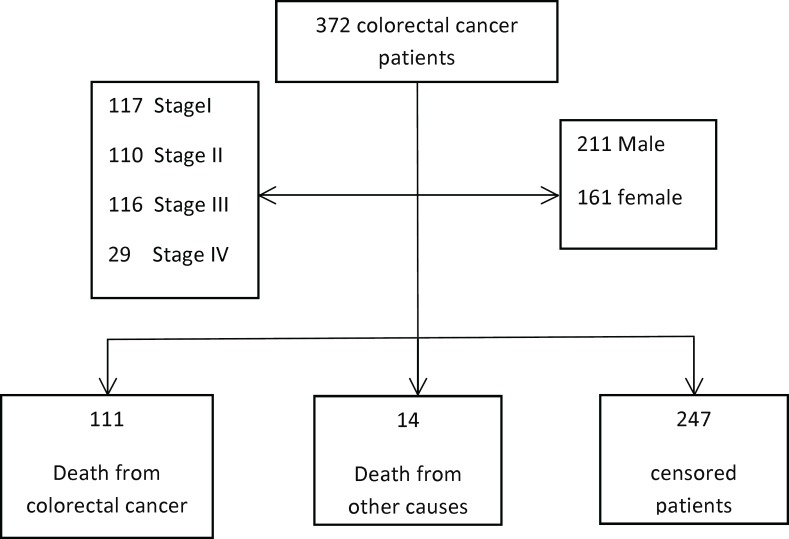
Flowchart of 372 colorectal cancer patients

 Overall, 372 CRC patients were included in the analysis, 211(56.7%) patients were male and 161 (43.3%) were female. 117 (31.5%) of them were in stage I of disease, 110 (29.6%) were in stage II, 116 (31.2%) were in stage III, and (29)7.8% of cases were in stage IV (Table 1 and Figure 1).

 111 (29.8%) of cases with CRC died due to colorectal cancer, 14 (3.8%) of them died from other causes of death, and 247 (66.4%) of them were survived until the end of the study. The mean±sd of age at diagnosis was 52.69±14.39 years (with range 12-84 years). The average BMI was 24.61±3.98. The mean±sd of survival time for subjects with colorectal cancer was calculated 62.05±48.78 with median=48 months (with P25:18, P75:103).

**Figure2:**
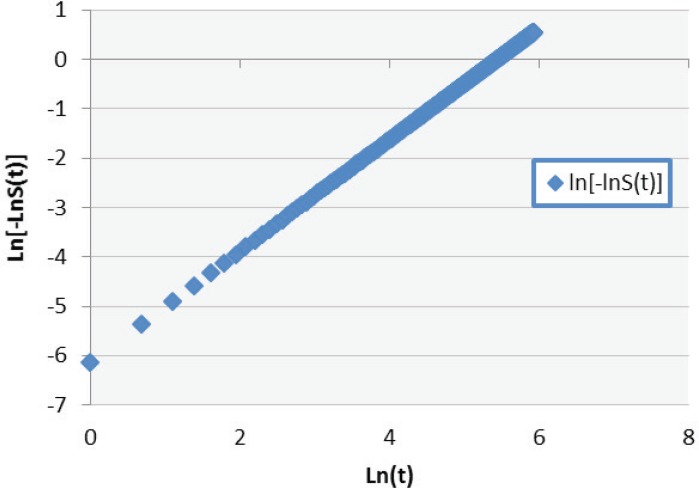
Negative Log of Negative Log Survivor Function Estimates.

The graph of the log–log survival against the log of failure time followed a linear trend which indicates that Weibull model is appropriate for this data (Figure 2). 

Results of survival analysis using a Weibull model, with considering competing risks and without it are shown in table 2 and 3.

 Results of Weibull model without competing risks:

The results of Weibull model without competing risks are shown in table 2. In Weibull model without competing risks, only sex was also significant prognosis (HR, 0.766; 95% CI, 0.599-0.978 [P<0.04] ). Other variables such as tumor stage, BMI and age at diagnosis had no impact on colorectal cancer mortality.

 Results of Weibull model with competing risks:

 The results of Weibull competing risks model with both Causes of Death are shown in table 3. There are some important differences between the covariate effects on the two competing events. Sex had no impact on colorectal cancer mortality. The effect of sex was statistically non-significant on both events (p=0.68 and p=0.52 for mortality of colorectal cancer and other causes, respectively). 

 Also the age at the time of diagnosis was not statistically significant for mortality of colorectal cancer. In contrast age at diagnosis had an impact on deaths due to other causes and the hazard ratio of age at diagnosis for these patients was 1.01(95% CI, 1.002-1.020) .

**Figure 3:**
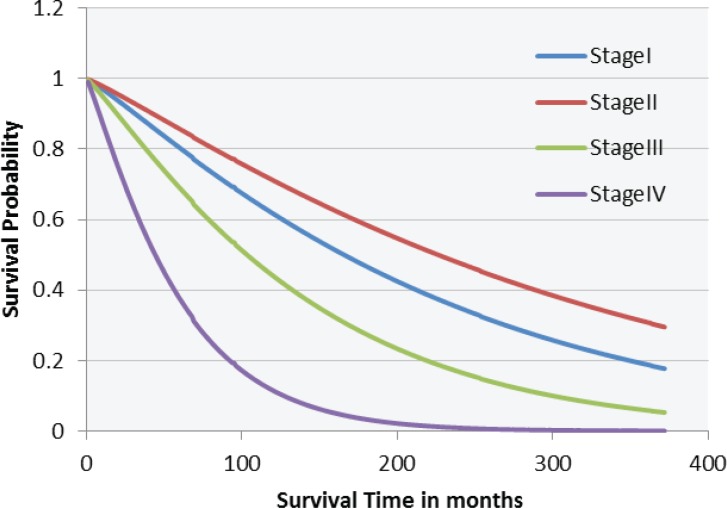
Estimated survival curves for stage I, stage II, stage III and stage IV patients with colorectal cancer based on a Weibull Competing risk model

 On Weibull competing risks model analysis of patients with stage I to IV disease was associated with an increased risk of death for both competing events [death from colorectal cancer and death from other causes]. (Stage IV: HR, 4.51; 95% CI, 2.91-6.99 [P<0.001]), while pathologic stage (stages III and IV) were associated with high hazards of colorectal cancer-related mortality (Table 3). Five-year CRC specific survival by stage was 0.81% for stage 1, 85% for stage 2, 69% for stage 3 and 37% for stage 4 (Figure 3). Also the impact of BMI was statistically significant for both events (P<0.001).

## Discussion

 In this study the association between survival of patients with colorectal cancer and prognostic factors were assessed using parametric models and also the survival time of colorectal patients were obtained. In these data, there were five different failures, including: dying due to CRC cancer, dying due to myocardial infarction, dying by stomach cancer or kidney and lung disease.

Results from competing risk analysis with the Weibull model indicated that just BMI and cancer stage (stage III, stage IV) are the prognosis factors of CRC survival in patients under study. These predictors were either significant in the Weibull model without considering competing risks, however tumor size and sex were also significant.

**Table1:** Description characteristics discrete values of the studied population (n=372)

**Covariate**	**Number (%)**	**Death due to CRC**	**Death due to other risks**	**Mean of survival time(se)**
Sex				
Man	211(56.7)	64(30.3)	7(3.3)	63.34(3.52)
Woman	161(43.3)	47(29.2)	7(4.3)	60.35(3.59)
Stage				
Stage I	117(31.5)	32(27.4)	2(1.7)	73.63(4.35)
Stage II	110(29.6)	19(17.3)	7(6.4)	64.5(4.81)
Stage III	116(31.2)	40(34.5)	3(2.6)	53.30(4.38)
Stage IV	29(7.8)	20(69)	2(6.9)	41(8.03)

**Table 2:** Result of the Weibull model analysis of 372 colorectal cancer patients without competing events analysis

**Covariate**	**Coefficient (SE)**	**HR**	**95% CI**	**P value**
BMI (Kg/m²)	0.004(0.003)	1.004	(0.996-1.011)	0.660
Age at diagnosis	0.002(0.001)	1.002	(0.998-1.005)	0.740
Sex				
Man		1		
Woman	-0.266(0.124)	0.766	(0.599-0.978)	0.040
Stage				
Stage I				
Stage II	-14.458(98.833)	1$\times 10^{-6}$	(0-7$\times 10^{20}$)	0.880
Stage III	-18.205(598.152)	2$\times 10^{-7}$	-	0.970
Stage IV	-12.893(87.005)	3$\times 10^{-6}$	(0-3$\times 10^{15}$)	0.900

**Table 3:** Results of the Weibull competing risks model analysis of 372 colorectal cancer patients with two competing events: death from colorectal cancer and death from other causes

**Event**	**Covariate**	**Coefficient (SE)**	**HR**	**95% CI**	**P value**
**Death from colorectal cancer**	BMI	-0.033(0.004)	0.968	(0.96-0.975)	<0.001
	Age at diagnosis	0.0005(0.002)	1.0003	(0.977-1.004)	0.342
	Sex				
	Man		1		
	Woman	-0.054(0.125)	0.947	(0.741-1.21)	0.681
	Stage				
	Stage I		1		
	Stage II	-0.352(0.229)	0.703	(0.449-1.103)	0.122
	Stage III	0.529(0.158)	1.698	(1.246-2.315)	<0.001
	Stage IV	1.507(0.224)	4.511	(2.911-6.992)	<0.001
**Death from other causes**	BMI	-0.031(0.011)	0.969	(0.949-0.99)	0.003
	Age at diagnosis	0.011(0.005)	1.011	(1.002-1.020)	0.010
	Sex				
	Man		1		
	Woman	-0.280(0.378)	0.756	(0.360-1.585)	0.522
	Stage				
	Stage I		1		
	Stage II	1.383(0.378)	3.986	(1.900-8.364)	<0.001
	Stage III	0.757(0.577)	2.132	(0.687-6.612)	0.201
	Stage IV	2.038(2.882)	7.675	(1.919-30.691)	0.004

This study indicated pathologic stage and BMI as the prognosis factors, using a Weibull model with competing risks analysis, while using the Weibull model without considering the competing events leads to significant predictors which may be due to over-estimation. This difference reflected the fact that ignoring the mortality which occurred due to other causes of death would be a potential source of over-estimation (14). Also, the confidence intervals for Weibull competing risk model were shorter compared to the confidence intervals for Weibull model without competing risks. So the Weibull competing risk model is more accurate.

In our study and based on a Weibull competing risk model, advanced stage disease (stage III, stage IV) had a greater impact on survival. The survival of patients with stage I was almost 4 times less, which is in line with results of others (15).

In our study, the crude median survival time for CRC patients was 48 months (4 years). A study in Iran in 2015 reported that the median survival time after CRC diagnosis was 3.5 years (7). In another study, median survival for patients with stage IV disease was 17 months (16).

 The 5-year survival rate following resection was 81% for stage 1, 85% for stage 2, 69% for stage 3 and 37% for stage 4. These figures can be compared with the results from USA (93.3% for stage 1, 82.5% for stage 2, 59.5% for stage 3 and 8.1% for stage 4) (17.)

The variation in survival for the same cancer among different countries is well known and depends upon the cancer registry, stage at presentation, access to medical care, availability of care and screening protocol (18). 

BMI had statistically significant effect on survival time; So that the hazard ratio of BMI for these patients is 0.968 which means that decreasing BMI would increase the risk of patient’s death due to colorectal cancer, while the effect of BMI was statistically non-significant on Weibull model without competing events. It is in contrary with Nilson’s study, who didn’t find any relationship between BMI and risk of colorectal cancer in their research (19).

Age at diagnosis was a significant predictor of a patient’s survival according to all models. As age increased, the rate of mortality increased. Mortality after colorectal cancer treatment may be associated with age, although evidence for this is conflicting (20.^21^). This finding is in line with the same study which reported age as the prognosis for CRC (22,23)

Sex was not significance according to competing risk model. In most countries, incidence and mortality rates are considerably higher in men than in women (24). Several studies reported superior survival in females (25,26); while, other studies did not report any differences, (27) which is similar to our results. 

 One of the limitations of this study is lack of accessing to some information, such as the number of metastasis site, grade, etc, which could have important effects on the survival rate of patients with colorectal cancer. Changing addresses and phone numbers for follow up were other limitations of this study. In future studies, this information will be included in competing risks survival for better prediction. Also, using other parametric distributions such as generalized Weibull which leads to cover different types of hazard functions is also suggested.

## References

[1] Dolatkhah R, Hossein Somi M, Jabbarpour Bonyadi M, Asvadi Kermani I, Farassati F, Dastgiri S (2015). Colorectal cancer in Iran: Molecular epidemiology and screening strategies. J Cancer Epidemiol.

[2] Li C, Lu HJ, Na FF, Deng L, Xue JX, Wang JW (2013). Prognostic role of hypoxic inducible factor expression in non-small cell lung cancer: a meta-analysis. Asian Pac J Cancer Prev.

[3] American Cancer Society (2015). Cancer Facts & Figures 2015.

[4] Azeem S, Gillani SW, Siddiqui A, Jandrajupalli SB, Poh V, Syed SS (2015). Diet and colorectal cancer risk in Asia--A systematic review. Asian Pac J Cancer Prev.

[5] Alwan A (2011). Global status report on noncommunicable diseases 2010.

[6] Ahmadi A, Mosavi-Jarrahi A, Pourhoseingholi MA (2015). Mortality determinants in colorectal Ccancer patients at different grades: A prospective, Cohort Study in Iran. Asian Pac J Cancer Prev.

[7] Dolatkhah R, Somi MH, Kermani IA, Ghojazadeh M, Jafarabadi MA, Farassati F (2015). Increased colorectal cancer incidence in Iran: a systematic review and meta-analysis. BMC Public Health.

[8] Pourhoseingholi MA, Zali MR (2012). Colorectal cancer screening: Time for action in Iran. World J Gastrointest Oncol.

[9] Baghestani AR, Gohari MR, Orooji A, Pourhoseingholi MA, Zali MR (2015). Evaluation of parametric models by the prediction error in colorectal cancer survival analysis. Gastroenterol Hepatol Bed Bench.

[10] Smith EC, Ziogas A, Anton-Culver H (2013). Delay in surgical treatment and survival after breast cancer diagnosis in young women by race/ethnicity. JAMA Surg.

[11] van Walraven C, McAlister FA (2016). Competing risk bias was common in Kaplan–Meier risk estimates published in prominent medical journals. J Clin Epidemiol.

[12] O’Connell J, Maggard M, Clifford K (2004). Colon cancer survival rates with the new American joint committee on cancer sixth edition staging. J Natl Cancer Inst.

[13] Kleinbaum DG, Klein M, Gail M, Krickeberg K, Samet JM, Tsiatis A, Wong W (1996). Parametric Survival Models. Survival Analysis.

[14] Belot A, Abrahamowicz M, Remontet L, Giorgi R (2010). Flexible modeling of competing risks in survival analysis. Stat Med.

[15] Haidinger G, Waldhoer T, Hackl M, Vutuc C (2006). Survival of patients with colorectal cancer in Austria by sex, age, and stage. Wien Med Wochenschr.

[16] Kumar S, Burney IA, Zahid KF, Souza PCD, Belushi MA, Meki TDMWA (2015). Colorectal cancer patient characteristics, treatment and survival in Oman–A single center Study. Assian Pac J Cancer Prev.

[17] O’Connell J, Maggard M, Clifford K (2004). Colon cancer survival rates with the new American joint committee on cancer sixth edition staging. J Natl Cancer Inst.

[18] Gatta G, Capocaccia R, Sant M, Bell CM, Coebergh JW, Damhuis RA (2000). Understanding variations in survival for colorectal cancer in Europe: A EUROCARE high resolution study. Gut.

[19] Nilsen TI, Vatten LJ (2001). Prospective study of colorectal cancer risk and physical activity, diabetes, blood glucose and BMI: exploring the hyperinsulinaemia hypothesis. Br J Cancer.

[20] Morris EJ, Sandin F, Lambert PC, Bray F, Klint A, Linklater K (2011). A population-based comparison of the survival of patients with colorectal cancer in England, Norway and Sweden between 1996 and 2004. Gut.

[21] Forman D (2009). Cancer Incidence and Survival by Major Ethnic Group, England, 2002–2006.

[22] Møller H, Sandin F, Robinson D, Bray F, Klint Å, Linklater KM (2012). Colorectal cancer survival in socioeconomic groups in England: variation is mainly in the short term after diagnosis. Eur J Cancer.

[23] Li XP, Xie ZY, Fu YF, Yang C, Hao LP, Yang LM (2013). Colorectal cancer concealment predicts a poor survival: a retrospective study. Asian Pac J Cancer Prev.

[24] Brenner H, Hoffmeister M, Arndt V, Haug U (2007). Gender differences in colorectal cancer: implications for age at initiation of screening. Br J Cancer.

[25] McArdle CS, McMillan DC, Hole DJ (2003). Male gender adversely affects survival following surgery for colorectal cancer. Br J Surg.

[26] Paulson EC, Wirtalla C, Armstrong K, Mahmoud NN (2009). Gender influences treatment and survival in colorectal cancer surgery. Dis Colon Rectum.

[27] Koo JH, Leong RW (2010). Sex differences in epidemiological, clinical and pathological characteristics of colorectal cancer. J Gastroenterol Hepatol.

